# OligoRAP – an Oligo Re-Annotation Pipeline to improve annotation and estimate target specificity

**DOI:** 10.1186/1753-6561-3-S4-S4

**Published:** 2009-07-16

**Authors:** Pieter BT Neerincx, Han Rauwerda, Haisheng Nie, Martien AM Groenen, Timo M Breit, Jack AM Leunissen

**Affiliations:** 1 Laboratory of Bioinformatics, Wageningen University and Research centre (WUR), P.O. Box 569, 6700 AN Wageningen, The Netherlands; 2 Micro array Department (MAD), Swammerdam Institute for Life Sciences, University of Amsterdam (UvA), Kruislaan 318, 1098 SM Amsterdam, The Netherlands; 3 Animal Breeding and Genomics Centre, Wageningen University and Research centre (WUR), P.O. Box 338, 6700 AH, Wageningen, The Netherlands

## Abstract

**Background:**

High throughput gene expression studies using oligonucleotide microarrays depend on the specificity of each oligonucleotide (oligo or probe) for its target gene. However, target specific probes can only be designed when a reference genome of the species at hand were completely sequenced, when this genome were completely annotated and when the genetic variation of the sampled individuals were completely known. Unfortunately there is not a single species for which such a complete data set is available. Therefore, it is important that probe annotation can be updated frequently for optimal interpretation of microarray experiments.

**Results:**

In this paper we present OligoRAP, a pipeline to automatically update the annotation of oligo libraries and estimate oligo target specificity. OligoRAP uses a reference genome assembly with Ensembl and Entrez Gene annotation supplemented with a set of unmapped transcripts derived from RefSeq and UniGene to handle assembly gaps. OligoRAP produces alignments of each oligo with the reference assembly as well as with unmapped transcripts. These alignments are re-mapped to the annotation sources, which results in a concise, as complete as possible and up-to-date annotation of the oligo library. The building blocks of this pipeline are BioMoby web services creating a highly modular and distributed system with a robust, remote programmatic interface.

OligoRAP was used to update the annotation for a subset of 791 oligos from the ARK-Genomics 20 K chicken array, which were selected as starting material for the oligo annotation session of the EADGENE/SABRE Post-analysis workshop. Based on the updated annotation about one third of these oligos is problematic with regard to target specificity. In addition, the accession numbers or ids the oligos were originally designed for no longer exist in the updated annotation for almost half of the oligos.

**Conclusion:**

As microarrays are designed on incomplete data, it is important to update probe annotation and check target specificity regularly. OligoRAP provides both and due to its design based on BioMoby web services it can easily be embedded as an oligo annotation engine in customised applications for microarray data analysis. The dramatic difference in updated annotation and target specificity for the ARK-Genomics 20 K chicken array as compared to the original data emphasises the need for regular updates.

## Background

DNA microarray technology has evolved rapidly to become the most popular platform for high throughput gene expression analysis as it allow biologists to measure the expression of entire transcriptomes at relatively high speed and low cost. This makes microarrays ideal for applications like sample clustering/fingerprinting, genome annotation, detection of differential gene expression, detection of polymorphisms and re-sequencing [1,2]. Microarrays contain oligonucleotides (probes) that can hybridise with the labelled reverse complement of mRNA. Since the probes are immobilised on the surface of an array and it is known which probes are located where on the array, signal at a certain spot can be used as a measure for gene expression. This requires that probes are unique for their target genes and hence optimal microarray design requires 1) a completely sequenced reference genome, 2) complete annotation for this reference genome to know what parts may be expressed and 3) complete knowledge about the natural variation amongst the sampled individuals.

Unfortunately there is currently not a single species for which such complete information is available. Although some reference genomes are now close to completion, annotation of these reference genomes as well as information on how individuals differ from these reference genomes is far from complete. Hence, microarray design is currently sub-optimal even for species with a rather complete reference genome. Probe design based on incomplete or erroneous data can lead to serious problems like non-specific probes causing cross hybridisation, orphan probes designed for non-existing targets, missing probes and misleading probes due to erroneous annotation.

Therefore, it is important to update the annotation for arrays regularly to improve the functional annotation of the targets as well as the reliability of probe-target assignments. Several tools have been developed for this purpose [3-12], but these provide either limited annotation, require complicated local installations with many dependencies, do not scale well or do not support our species of interest. We have developed OligoRAP (Oligo ReAnnotation Pipeline) to overcome these issues.

## Implementation

**Figure 1:**
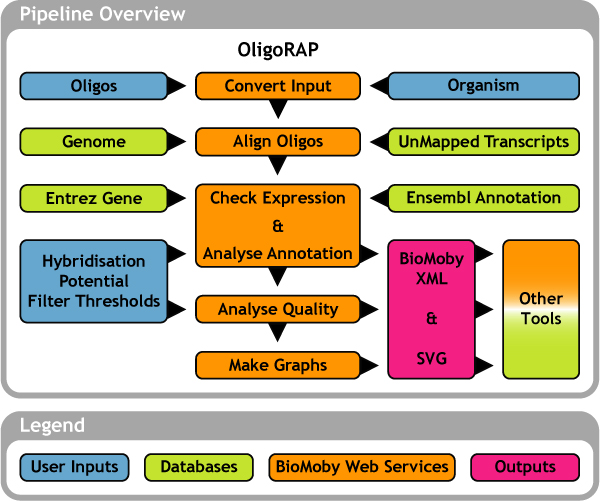
**Summarizing OligoRAP flowchart**. Blue blocks represent user input, green blocks databases, pink blocks output and finally orange blocks represent one or more BioMoby web services. For a more detailed description see Additional files 1, 2, 3, 4, 5, 6.

The pipeline consists of 5 steps: I. Convert oligo library data into BioMoby objects, II. Align oligos with a reference genome assembly and with a set of unmapped transcripts (UMTs), III. Analyse oligo annotation, IV. Analyse oligo quality and V. Make summary charts (see Figure 1). Implementation details are described and illustrated in Additional files 1, 2, 3, 4, 5, 6. In this section we will only focus on the key advantages of OligoRAP.

Firstly, OligoRAP does not rely solely on a reference genome or solely on transcripts (or sequences derived thereof), but uses both where possible. For the genome OligoRAP uses reference assemblies and annotation as provided by the Ensembl [13] project. Ensembl was chosen as primary annotation source, because it is the largest and richest resource of its kind with support for most popular model species in the animal kingdom. In addition to reference assemblies OligoRAP uses a set of unmapped transcripts (UMTs) to get a more complete picture. The UMT set contains RefSeq [14] and UniGene [14] entries, which failed to map to the reference assembly. Where available annotation derived from Ensembl (for hits on the genome) and from RefSeq or UniGene (for hits on UMTs) can be expanded with links to Entrez Gene [14] and GO [15]. The combination of reference genome supplemented with UMTs provides optimally complete annotation for well-annotated species whilst keeping redundancy at a minimum. At the same time this strategy is flexible enough to support less well-annotated species even if there is no reference assembly available. In that case all of a species' transcripts simply become part of the UMT set.

Secondly, OligoRAP provides annotation for all hits instead of only for the best hit. This allows OligoRAP to provide not only updated annotation, but also oligo target specificity based on the amount and type of hits. OligoRAP can differentiate between primary hits (high hybridisation potential) and secondary hits (low hybridisation potential). Hybridisation potential is determined using three filters, which users can adjust based on their experimental setup. Based on their target specificity oligos are divided into six target specificity classes (TSCs): 1. Gene-specific probes with maximum signal potential, 2. Gene-specific probes with reduced signal potential, 3. Non-specific probes with maximum signal potential, 4. Non-specific probes with mixed signal potential, 5. Non-specific probes with reduced signal potential and 6. Orphan probes with background signal potential.

Finally, each of the steps is implemented as one or more web services [16], which were built using the BioMoby framework [17,18]. These web services provide remote programmatic access and can be glued together using a variety of BioMoby clients like the Taverna Workbench [19] or custom code built with the BioMoby Perl or Java framework. Using web services we created a highly customisable and modular annotation pipeline with a robust interface. This allows for OligoRAP to be embedded in microarray data analysis workflows for improved scalability without tedious, local installations suffering from complex dependencies.

## Results and discussion

**Figure 2:**
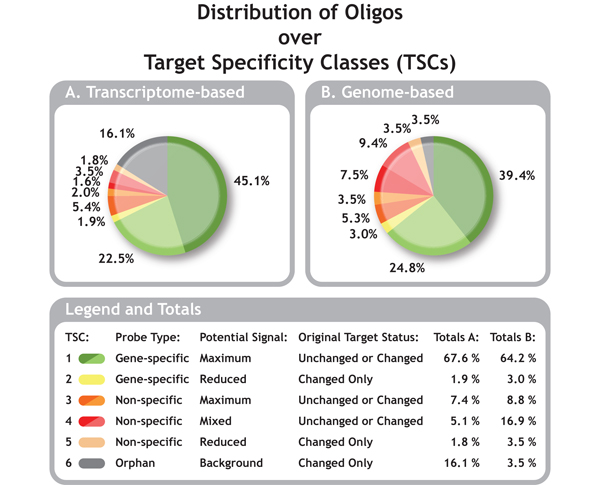
**Distribution of Oligos over Target Specificity Classes (TSCs)**. Distribution of the 791 oligos selected for the workshop over the 6 TSCs with transcriptome-based (A) and genome-based target specificity (B). The status of the link between the oligo and the accession number/identifier it was originally designed for is indicated by a tint difference in the colour for TSC 1, 3 and 4: accession/id still present in the annotation, hence "target unchanged" (dark tint) or accession/id absent, hence "target changed" (light tint). For TSC 2, 5 and 6 the target status is always "changed".

OligoRAP was used to update annotation and target specificity for the subset of 791 oligos from the ARK-Genomics 20 K chicken array (see methods in Additional file 1). Figure 2 shows how these oligos are divided over OligoRAP's target specificity classes (TSCs) with transcriptome-based target specificity (TbTS) in Figure 2A and genome-based target specificity (GbTS) in figure 2B.

### Transcriptome-based versus genome-based target specificity

Up till recently the transcriptome of higher eukaryotes was thought to contain a very small subset of the genome. For example in Ensembl 50 less than 5% of the chicken genome is annotated as exon. Since only potentially expressed sequences can hybridise to probes on a microarray, most oligo design and annotation efforts have focused on known and/or predicted transcripts without taking the rest of the genome into account. Apart from a few structural elements like the centromeres and telomeres it's still not clear what the function of the other 95% or more of DNA is, but slowly evidence is piling up indicating the size of the transcriptome is vastly underestimated. Especially the pilot phase of the ENCODE project showed that the human "genome is pervasively transcribed, such that the majority of its bases can be found in primary transcripts" [20]. It remains unclear whether all these transcripts are biologically functional or whether they just represent noise, but it is clear that all transcripts can potentially hybridise with the oligos on microarrays. Therefore it is probably more appropriate to evaluate target specificity in the context of the entire genome as compared to what is currently annotated as transcriptome.

Looking at TbTS and GbTS for the 791 ARK-Genomics chicken oligos the total amount of gene-specific oligos differs only by 2.3% with 69.5% and 67.2%, respectively. Hence taking the entire genome into account as compared to looking only at the transcriptome does not lead to a dramatic decrease of gene-specific probes. Unfortunately at least one third of the probes are non-specific. For these problematic non-specific probes the TbTS and GbTS pictures look quite different.

### Annotation quality

For most of the oligos it is extremely difficult to verify their predicted target specificity except for the orphan oligos of TSC 6. The 791 oligos selected as starting material for this EADGENE/SABRE workshop were picked, because they do show a high differential signal on the microarrays. Hence these oligos clearly bind labelled cDNA derived from one or more target genes, but OligoRAP classifies 3.5% and 16.1% of the oligos as orphans with GbTS and TbTS, respectively. These numbers indicate that OligoRAP's TSC assignments are currently more an indicator for the relatively immature status of the chicken genome assembly and its annotation than for target specificity.

Furthermore, for almost half of the oligos, the sequence identifier they were originally designed for is no longer present in their updated annotation, which is indicated with "target changed" in Figure 2. The fact that these identifiers no longer link to these oligos not necessarily means that the oligo no longer represents expression of the same gene as before, but it does indicate at least major changes in the annotation. On the other hand annotation associated with certain identifiers may have received considerable "minor" updates keeping the sequence identifier intact. Hence, the large amount of oligos with changed targets is still an underestimate of the total amount of changed annotation.

### Future work

Although the ENCODE pilot study covered only approximately 1% of the human genome it is clear that our view on the transcriptome will change dramatically over the next years. This will have a big impact on oligo annotation & target specificity making it more important than ever to be able to update oligo annotation quickly and regularly. In addition to regular updates of the data, annotation pipelines like OligoRAP will need to be updated too to adapt the annotation strategies to our changing insights in gene expression.

## Conclusion

Microarray probes are designed on incomplete data. Therefore it is important to update probe annotation and estimate target specificity regularly. OligoRAP provides such functionality for Ensembl species and can easily be embedded in customised applications for microarray data analysis due to its design based on BioMoby web services. The rather high amount of oligos with changed targets shows the importance of updated annotation and reflects the limited amount and quality of the annotation available at the time the ARK-Genomics 20 K chicken array was designed.

## Further information

ZIP-archive containing the final results of the OligoRAP pipeline run as well as all intermediate results. See included README for details.



## Competing interests

The authors declare that they have no competing interests.

## Authors' contributions

PBTN designed and programmed the pipeline of web services and drafted the manuscript. HR conceived the pipeline, participated in its design and helped to improve the manuscript. HN helped with data analysis for debugging and helped to improve the manuscript. TMB, MAMG and JAML secured funding, managed the project and helped to improve the manuscript. All authors read and approved the final manuscript.

## Supplementary Material

Additional file 1 **Implementation details, Availability & Requirements and Methods**. Text describing details with regard to Implementation, Availability & Requirements and Methods. Click here for file

Additional file 2 **Detailed flowchart**. Figure in PDF format. (A) OligoRAP components. User inputs are in blue, databases in green and results in orange. Yellow blocks represent a single synchronous web service or a set of two asynchronous services for a specific task (one service for job submission and one for requesting a job's status). Some steps are executed multiple times. BLAT, BLAST, Concatenate, Analyse Annotation and Merge Hits and Analyse Quality are executed multiple times for multiple chunks as indicated by sets of three connecting lines starting with a filled circle (●). The Create Chart step is executed multiple times for different inputs (not chunks) as indicated by a set of two connecting lines starting with a filled square (■). (B) Some examples of how OligoRAP can be extended or linked to downstream analyses tools. Click here for file

Additional file 3 **Relationships between filter thresholds, primary & secondary hits and estimated signal intensity**. Figure in PDF format. Primary hits (green) represent (near) perfect alignments of oligos with their targets. Secondary hits (orange) are defined as worse than primary hits, but still capable of generating signal above background. Relative signal intensity is shown on the vertical axis and the 3 filters – mismatches, sequence identity and longest contiguous stretch – on 3 horizontal axes. Signal intensity drops as the amount of mismatches increases and as the percentage sequence identity or the length of the longest contiguous stretch decreases. Estimated signal intensity above the primary hit threshold (green) is defined as "maximum signal". Estimated signal below the primary and above the secondary hit threshold (orange) is defined as "reduced". Finally estimated signal below the secondary hit threshold is defined as "background signal". Click here for file

Additional file 4 **Overview of OligoRAP's six target specificity classes, which are defined by the amount of primary and secondary hits**. Figure in PDF format showing how target specificity classes are defined based on hits. Primary hits (green) represent (near) perfect alignments of oligos with their targets. Secondary hits (orange) are defined as worse than primary hits, but still capable of generating signal above background. Classes are named after the combination of probe type (gene-specific, non-specific or orphan) and estimated potential signal (maximum, reduced, mixed or background). Click here for file

Additional file 5 **OligoQualityAnalyser example output for Compugen oligo CGEN-MOUSE_30000003_1**. Figure in PDF format showing an example of OligoQualityAnalyser output with an OligoQualityRecord (blue) containing 2 hits/alignments (green) and 2 target specificity assignments (orange). The first hit overlaps with Ensembl features resulting in annotation in the form of cross-references (purple), while the second hit targets 'intergenic' space resulting in a lack of cross-references. Each target specificity block contains the oligo's specificity for two contexts – genome and transcriptome – and refers by id attribute to the filter settings (thresholds) described elsewhere in the XML (not shown). The Cigar Like Line (CLL) is derived from the Ensembl Cigar line [21] and is used to store alignment details (matches, mismatches, insertions, deletions & intron gaps) in a compact string. See figure additional file 6 for detailed CLL examples. Together with subject sequence accession/ID, start, and stop, the CLL provides all information necessary to reconstruct the alignments. It can be used for example to create UCSC "custom tracks" [22,23] for visualization in the UCSC or Ensembl genome browsers. Click here for file

Additional file 6 **Detailed CLL examples**. Figure in PDF format showing five example alignments with their accompanying cigar like lines (CLLs). A CLL is a compact way to represent the alignment of a first sequence (query) with a second one (subject/DB). In these examples the single stranded oligo is the query and the double stranded DNA the subject. Example with matches and substitutions of an oligo hit on the forward DNA strand (1). Similar example of an oligo hit on the reverse DNA strand (2). Note that the CLL describes the alignment from the perspective of the oligo in terms of insertions and deletions, but is always read from left to right with the forward strand of the subject written from 5' on the left to 3' on the right side. Hence in the case of example 2 the CLL corresponds to 3' on the left to 5' on the right for the oligo sequence. Examples of insertions & deletions (3) and of an intron gap (4). In this context introns are special cases of deletions and usually the result of merging multiple smaller hits into one larger alignment. Example of mixed case nucleotides (5): a number followed by 2 or more characters (m/s/i/d/n) indicates this amount of nucleotides can be a mix of the corresponding classes. In this case there are 25 ns nucleotides, which corresponds to a mix of substitutions with an intron gap. Due to the substitutions it's not possible to determine exactly where the intron gap starts and ends in the oligo sequence. Hence alignments corresponding to 7m5s20n9m, 7m3s20n2s9 m (shown) and 7m20n5s9 m can al be written as 7m25ns9m. Click here for file
