# Supplementary material for: OligoRAP – an Oligo Re-Annotation Pipeline to improve annotation and estimate target specificity
Source: BMC Proc. 2009 Jul 16;3(Suppl 4):S4. doi: 10.1186/1753-6561-3-S4-S4 (PMC2712747; doi:10.1186/1753-6561-3-S4-S4)
Supplement: Additional file 1 — Implementation details, Availability & Requirements and Methods. Text describing details with regard to Implementation, Availability & Requirements and Methods. [file 1753-6561-3-S4-S4-S1.pdf]

Additional file 1

Implementation details

OligoRAP consists of 5 steps as depicted in figure 1: I. Convert oligo library data into BioMoby objects, II. Align oligos with a reference genome assembly and with a set of unmapped transcripts (UMTs), III. Analyse oligo annotation, IV. Analyse oligo quality and V. Make charts. These steps are described here in detail followed by more information on the web service interfaces in general. For a more detailed flowchart see figure Additional file 2.

I. Converting oligo library data into BioMoby objects

The oligos are divided into chunks in order to distribute the load and be able to re-do subsets of the analysis in case something goes wrong. In case the oligo sequences are not available as chunks of BioMoby multi sequence FASTA objects, the Tab2MultiSeqFastaChunks or Fasta2MultiSeqFastaChunks services can be used to convert from tab delimited or FASTA format respectively. In addition to the oligo sequences the systematic name of the organism for which to annotate the sequences is required and together these inputs are forwarded to the next services.

II-A. Aligning oligos with a reference genome assembly

Firstly, the MobyBlat service is executed to align the oligos with a reference genome. MobyBlat uses the BLAST Like Alignment Tool (BLAT) [1], which uses an in memory index to provide a huge query speed improvement over BLAST although at the expense of steeper memory requirements. BLAT parameters are optimised to minimise the risk of missing short relevant hits due to intron gaps, while keeping the server's memory requirements below 2 GB (see methods). A disadvantage of BLAT is that it might miss hits to highly redundant parts of the genome, because it stores only unique or relatively rare fragments (tiles) in its index.

Secondly, oligos without any BLAT hit are aligned with the MobyBlast service, which uses NCBI BLAST [2, 3]. BLAST searches are executed without the default low complexity region filtering to detect hits for oligos that target conserved domains in members of large gene families or for oligos that were accidentally designed for repeats (see methods for additional parameters).

Since the amount of oligos without BLAT hit is usually less than 3%, this strategy provides an ideal combination of speed and accuracy. Once both MobyBlat and MobyBlast have completed a chunk, their results are passed on to the ConcatenateFiles service, which simply appends MobyBlast results to those of MobyBlat.

II-B. Aligning oligos with unmapped transcripts (UMTs)

A set of UMTs is used in addition to a reference assembly, because no single assembly is 100% complete. This UMT set contains all RefSeq [4] RNA accessions as well as UniGene clusters that were not mapped on the genome by Ensembl [5] with high sequence identity (see construction of UMT sets below). Small imperfections are allowed in the Ensembl alignments to prevent accessions from being included into the UMT dataset due to natural variation amongst individuals of a species.

Similar as for the alignments with the genome the oligos are first aligned with the UMT set using MobyBlat and only oligos without any BLAT hit are aligned using MobyBlast. Since UMTs don't contain introns, there is no need to increase BLAT sensitivity to pick up short hits to intron-separated exons. Other parameters were the same as for the alignments with the genome assembly (see methods). Just as for alignments with the genome, results from MobyBlat and MobyBlast are combined per chunk with the ConcatenateFiles service.

Construction of UMT sets

For each OligoRAP supported species, the sets of unmapped transcripts (UMTs) contain all RefSeq [4] RNA accessions of that species that were not mapped on the genome by Ensembl [5] with 97% sequence identity or better and are based on experimental evidence (accession numbers starting with "NR" or "NM"). Since RefSeq does not include records for (clustered) transcript fragments if there is no evidence for a complete CDS and oligos are frequently designed based on ESTs, relying solely on RefSeq for UMTs would be needlessly incomplete. Therefore, member sequences of UniGene clusters [4] are also included in the UMT set if none of a cluster's member sequences was aligned with the genome by Ensembl at 97% sequence identity or better and if the cluster didn't contain a RefSeq accession. Finally, RefSeq RNAs and UniGene clusters are only included in the UMT set if they were "known" at the time an Ensembl data set was generated to prevent redundancy due to new sequences, which were simply not yet taken into account. This means RefSeq RNAs and UniGene clusters must exist either as "DNA align feature" and/or "Xref" and/or "unmapped object" in the Ensembl databases. This procedure is necessary, as information on which versions of external databases like RefSeq and UniGene were used for a certain Ensembl release tends to be inconsistent and/or incomplete.

III. Analysing oligo annotation: parsing alignments and fetching annotation for individual hits

The concatenated alignment reports are then sent to the OligoAnnotationAnalyser web service, which uses BioPerl [6] for NCBI BLAST XML parsing.

For hits on the genome OligoAnnotationAnalyser checks if a hit overlaps any sequence features annotated by Ensembl, that indicate this hit might be expressed. This "expression evidence" includes amongst others: Ensembl Gene IDs, Ensembl Transcript IDs, INSDC (DDBJ & EMBL & GenBank) RNA/cDNA accessions including full length sequences as well as fragments like ESTs, UniProt KB accessions, UniGene accessions and RefSeq RNA accessions (see annotation retrieval below for details). These features range from de novo in silico gene predictions to well-known, manually curated proteins, but OligoAnnotationAnalyser does not take reliability of the "expression evidence" into account. Accession numbers of the overlapping sequence features are added as cross-references to the corresponding oligo hit and as long as there is at least one such a cross-reference, hits are flagged as potentially expressed. Based on Ensembl Gene IDs, the cross-references for a hit can be expanded with Gene Ontology (GO) term IDs [7] and Ensembl homologs (see annotation retrieval below for details).

After checking for expression evidence OligoAnnotationAnalyser performs an "intron-check" to see if there were any partial hits that together might represent a complete match. Such partial hits are merged into a single hit if two or more consecutive hits share at least one or more transcription cross-references.

For hits on the UMT set OligoAnnotationAnalyser simply adds the accession number of the UMT as cross-reference and flags the hit immediately as potentially expressed. Redundant hits to members of the same UniGene cluster are dropped, keeping only the first one of the best hits.

Next, for any hit on either genome or UMT set OligoAnnotationAnalyser uses mapping from Entrez Gene [4] to fetch Entrez Gene and GO term IDs based on RefSeq, UniGene and INSDC cross-references (see annotation retrieval below for details).

Finally OligoAnnotationAnalyser uses three filters to determine the hybridization potential of each hit: number of mismatches, longest contiguous stretch and percentage sequence identity. For each filter the client must specify thresholds depending on hybridization protocol, array type and array platform. Imperfect hits, whose hybridization potential is so weak they cannot be distinguished from background noise, are dropped. In an experiment for the empirical establishment of oligo probe design criteria He et al. have shown that a single filter couldn't detect all cross-hybridizing probes, but a combination of several filters could [8]. Therefore the filters in OligoAnnotationAnalyser are complementary. Hence, only if one or more filters predict hybridization potential for a hit it is preserved.

Annotation retrieval

Ensembl sequence features are retrieved from the Ensembl DNA align feature (DAF), protein align feature (PAF) or cross-reference (Xref) tables, which are part of the *core*, *cdna* (if present) and *otherfeatures* databases. We were able to reduce the query time for a complete oligo library from several weeks to several hours on our dedicated MySQL server by implementing speed optimizations involving partitioning of the data. This involves the creation of short "virtual contigs" (3000 nucleotides long) and remapping of features, which were originally assigned to complete chromosomes to these short virtual contigs. Due to the speed optimisations the Ensembl API cannot be used to fetch DAFs, PAFs and Xrefs. Raw SQL queries are used instead. In order for a DAF, PAF or an Xref to be scored as expression evidence these Ensembl features must align to the reference genome with 97% identity or better. This allows for small differences due to natural variation and/or sequencing errors. Furthermore an optional parameter can be used to shorten the region where the oligo aligned with the genome on both ends before checking if that hit overlaps any expression evidence. This prevents labelling hits as unexpressed due to small errors in gene models, small differences in alternative splicing or alignments that erroneously extend a few nucleotides into an intron. This optional trimming only affects the procedure that checks for overlapping Ensembl features and hence does not effect the alignment of the oligo with the genome in the final results. If there are not enough nucleotides available to trim maximally, hits are trimmed such that there will be at least 1 nucleotide left. DAF, PAF and Xref accession numbers are added as expression cross-references to the corresponding oligo hit.

Ensembl Gene IDs can be used to retrieve Gene Ontology (GO) term IDs [7] and Ensembl homologs from the Ensembl *go* and *compara* databases, respectively. For the latter clients can specify which homology types (as defined by Ensembl: ortholog_one2one, ortholog_one2many, ortholog_many2many, apparent_ortholog_one2one, between_species_paralog and within_species_paralog) and what model species to retrieve homologs for. GO term IDs are appended to hits as additional annotation cross-references whereas Ensembl homologs are appended as homology cross-references.

For alignments with UMTs, the accession number of the UMT is appended as transcription cross-reference to the corresponding oligo hit.

For any hit on either genome or UMT set OligoAnnotationAnalyser can fetch Entrez Gene IDs based on RefSeq, UniGene & INSDC (DDBJ & EMBL & GenBank) transcription cross-references based mapping provided by Entrez Gene [4]. The mapping is taken from the *gene2unigene*, *gene2refseq* and *gene2accession* as provided by the NCBI ftp-server (ftp://ftp.ncbi.nlm.nih.gov/gene/DATA/). Optionally if an Entrez Gene ID is available it may be used to link (additional) GO term IDs to the oligo hits using the *gene2go* file also available from the NCBI ftp-server. GO term IDs are appended to oligo hits as annotation cross-references, while Entrez Gene IDs are appended as additional transcription cross-references.

IV. Analysing oligo quality: estimating target specificity for each oligo and the library as a whole

The OligoAnnotationAnalyser results, containing the oligo hits and their annotation, are sent to the OligoMergeXML web service, which combines genome and UMT hits for each oligo per chunk. All merged chunks together are then sent to the OligoQualityAnalyser web service.

OligoQualityAnalyser uses the same three user-definable filters as OligoAnnotationAnalyser to determine the hybridization potential of each hit, but where OligoAnnotationAnalyser only checks if a hit can generate any signal above background noise, OligoQualityAnalyser differentiates between primary and secondary hits (figure additional file 3). Primary hits are defined as (near) perfect hits having optimal signal to noise ratio under the hybridization conditions. Imperfect hits, not qualifying as primary hit, but still causing a significant amount of noise are labelled secondary hits. Furthermore multiple combinations of filter settings can be provided in a single run to determine the effect of more lenient or more stringent settings on oligo target specificity. Just as in OligoAnnotationAnalyser the filters are complementary. So, if the filters predict different hybridization potentials for a hit it is labelled with the best hybridisation potential predicted.

OligoQualityAnalyser determines both oligo target specificity as well as the overall quality of the entire array. Based on the amount of primary and secondary hits the oligos are divided into six target specificity categories (TSCs) (figure additional file 4):

1. Gene-specific probes with maximum signal potential
2. Gene-specific probes with reduced signal potential
3. Non-specific probes with maximum signal potential
4. Non-specific probes with mixed signal potential
5. Non-specific probes with reduced signal potential
6. Orphan probes with background signal potential.

Gene-specific probes have only one hit, non-specific probes hit multiple targets and orphan probes no longer hit any targets. Maximum signal potential indicates primary hits, reduced signal potential indicates secondary hits, mixed signal potential is the result of a mix of both primary and secondary hits and finally background signal potential means the oligo doesn’t have any hits and hence is an orphan. When OligoQualityAnalyser determines target specificity it does so twice for different contexts: genome-based target specificity (GbTS) and transcriptome-based target specificity (TbTS). For GbTS all hits on genome and UMTs are taken into account irrespective of whether they have any evidence for potential expression or not, but for TbTS only hits with expression evidence are taken into account.

Usually a single hit for an oligo means it is target specific and multiple hits indicate potential cross-hybridization, but there are exceptions. RefSeq might for example contain redundancy as result of splice variants and UniGene clustering is not 100% perfect. Secondly, it is also possible that an oligo hits the same target more than once. Therefore, OligoQualityAnalyser counts multiple hits with the same Entrez Gene ID or the same Ensembl Gene ID cross-references as a single hit with the hybridization potential of the best one. On the other hand it is also possible for genes to overlap on the same strand. Therefore, hits on the same strand with cross-references to more than one Ensembl Gene ID are counted as multiple hits.

OligoQualityAnalyser keeps track of the amount oligos in each TSC per combination of filter settings. In addition to these summaries the results now contain for each oligo: 1) Oligo accession/ID and optionally the accession/ID of the original target the oligo was designed for, 2) Oligo length, 3) Zero or more hits on genome and/or UMTs with detailed alignment information and zero or more cross-references linking the hit to annotation, 4) Genome- and transcriptome-based target specificity class assignments per combination of filter thresholds. See figures additional file 5 and 6 for examples of OligoQualityAnalyser output and of the Cigar Like Lines (CLLs) used to store alignment details. OligoRAP's annotation consists of accession numbers and the database they belong to. This is a concise way of linking oligos to genes, transcripts and proteins, but mere accession numbers are not very meaningful to biologists, who are generally interested in attributes such as gene symbols, functional descriptions or GO terms. Retrieving all data for all accessions from all databases would generate a huge amount of data, which is not always useful and highly redundant. Therefore it was decided to store only the accession numbers as cross-references and leave it up to bioinformaticians to use the cross-references with downstream tools for their custom needs. Some example applications of how OligoRAP can be extended or used with additional tools are given in figure additional file 2-B, but this is beyond the scope of this paper.

V. Making charts

The summaries with the amount of oligos in each TSC are sent on to the MobyPieChart service, which generates several pie charts in SVG format. These pie charts provide an overview of the target specificity of all probes of the oligo-nucleotide library for a certain combination of filter settings.

Web services

Unfortunately, sending big datasets as part of the payload of a web service is problematic for most popular SOAP libraries (used for service execution). Therefore, we use "pass-by-reference", which means that an URL replaces results in case large output can be expected for a service (table 1). A client or the next service in the pipeline can then download and parse the data using alternative mechanisms. Although pass-by-reference works well as a workaround with the current BioMoby 1.0 standard, BioMoby has not yet standardised on a mechanism for pass-by-reference, but this will change soon as it is a key feature of Taverna 2 and the BioMoby community is actively discussing an extension to make it part of the standard.

Some of the OligoRAP services are asynchronous, because they can easily take more than the default timeout on the Internet (5 minutes) to do their job (table 1). In these cases functionality is divided over two services: one to submit a job returning a link to retrieve the results and one to request the job status. Asynchronous services

dispatch jobs internally to a small Grid Engine (GE) [9] cluster. Once a job has completed the results can be downloaded from the link provided by the submission service. Hence all asynchronous services use pass-by-reference.

Web service	Asynchronous	Uses pass-by-reference
Tab2MultiSeqFastaChunks	No	Yes
Fasta2MultiSeqFastaChunks	No	Yes
MobyBlat	No	Yes
MobyBlast	Yes	Yes
ConcatenateFiles	No	Yes
OligoAnnotationAnalyser	Yes	Yes
OligoMergeXML	No	Yes
OligoQualityAnalyser	Yes	Yes
MobyPieChart	No	No

Table 1 - OligoRAP web services, whether their execution is asynchronous and whether they use pass-by-reference.

Availability & requirements

Project name: OligoRAP
Project home page: https://www.bioinformatics.nl/phenolink/home/

The web services were designed for remote programmatic access, which is freely available for all within limits of our computing facilities. (Restrictions on use by non-academics: BLAT used in the MobyBlat service requires a license for commercial use and hence access to MobyBlat requires a license for commercial use too. The pipeline can be used without MobyBlat without any loss of functionality although at the expense of a much longer runtime.) All services are registered in the public BioMoby Central repository under service authority www.bioinformatics.nl. Remote programmatic access only requires a BioMoby compatible client.

The web services of the pipeline have many dependencies making them unsuitable for easy portability. Source code is nevertheless available upon request from the authors. The underlying databases are currently updated for even numbered Ensembl releases and OligoRAP supports Bos taurus, Danio rerio, Homo sapiens, Gallus gallus, Mus musculus and Rattus norvegicus. Other Ensembl species can be indexed on request.

Clients:

Operating system(s): platform independent / various depending on client.
Programming language: various depending on client.
Other requirements: internet access, some clients require the BioMoby Perl and/or Java framework available from www.biomoby.org.
License: various

Web services:

Operating system(s): Linux
Scripting language: Perl

Other requirements: BLAT, BLAST, Ensembl databases, Entrez Gene database, RefSeq database, UniGene database, BioMoby Perl modules, BioPerl, many Perl modules available from CPAN (dependencies for the former two), Saxon-B, Java, MySQL, Apache and Grid Engine (GE)
License: GNU GPL v2

Methods

Updating annotation

All web services were running on servers with SuSE Linux Enterprise Server (SLES) 9. Asynchronous services dispatched jobs to a 10 node SLES 9 cluster with Grid Engine 6u10. The genome assembly (WASHUC2) was downloaded from Ensembl. UMT sequences were derived from RefSeq 30 and UniGene Gga 40. Expression evidence for oligo hits was derived from the Ensembl 50. Mapping from UniGene, RefSeq and INSDC (DDBJ & EMBL & GenBank) to Entrez Gene was taken downloaded from the NCBI ftp server on 26-08-2008. GO terms were derived from both the Ensembl 50 as well as from the Entrez Gene d.d. 26-08-2008. Databases were stored on local servers running SLES 9 with MySQL 4.1.

Web service specifics

MobyBlat: BLAT suite version 34 [1]; gfServer: tileSize = 12 nucleotides, minMatch = 1 tile, stepSize = 6 nucleotides; gfClient - all alignments: q = rna, gfClient - additional parameters only for alignments with a genome assembly: maxIntron = 1.000.000 nucleotides, minScore = 0; Other optional parameters: out = blast7 (BLAST XML).
MobyBlast: NCBI BLAST 2.2.13 [2, 3]; blastall: p = blastn, m = 7, S = 3 for alignments with the genome assembly or S = 1 for alignments with UMT sequences, F = F.
OligoAnnotationAnalyser: BioPerl 1.5.2 [6] except for blastxml.pm, for which we used an outdated version from CVS (10-05-2007) as later releases were either broken or didn't scale well. (This problem should be fixed now and recent versions of BioPerl can be used again with recent versions of BLAST (Personal communication with BioPerl developer Chris Fields.)); Thresholds for dropping hits: mismatches = 45, contiguous stretch = 16, percentage identity = 30. Optional parameters: fetch_go_from_ensembl = 1, fetch_go_from_Entrez Gene = 1, ortholog_one2one = 1, apparent_ortholog_one2one = 0, ortholog_one2many = 1, ortholog_many2many = 1, between_species_paralog = 0, within_species_paralog = 0, fetch_ensembl_bos_taurus_homolog = 1, fetch_ensembl_gallus_gallus_homologs = 1, fetch_ensembl_homo_sapiens_homologs = 1, fetch_ensembl_mus_musculus_homologs = 1, fetch_ensembl_rattus_norvegicus_homologs = 1, expression_evidence_overlap_mismatch = 5.
OligoQualityAnalyser hybridisation filter thresholds (primary/secondary): mismatches* = 1/2; longest contiguous stretch* = 55/20; percentage identity* = 95/85; * = Filter thresholds derived from He et alia [8] except for the mismatches filter which is basically disabled with these thresholds to make the OligoRAP filters as close as possible similar to those of two other oligo annotation pipelines for a comparison of annotation strategies described elsewhere in this issue.
MobyPieChart: Uses Saxon-B 8.8J from Saxonica [10] with an XSLT to transform the BioMoby XML into SVG; Optional parameters: three_dimensional = 1,

throw_shadow = 1, series_clustering_threshold = 1, clustered_series_label = Others, clustered_series_color = #AAAAAA.

Microarray

The microarray used is the ARK-Genomics Chicken 20K array consisting of 20.460 probes ranging in length from 60 to 75 nucleotides with the majority of the probes 70 nucleotides long [11]. It was designed in 2005 based on: 1) INSDC (DDBJ/EMBL/GenBank) ESTs/cDNAs including the UMIST ChESTs, 2) Ensembl 30 with gene models based on various sources ranging from highly reliable chicken UniProtKB/Swiss-Prot proteins to relatively unreliable *ab initio in silico* gene predictions, 3) miRBase micro RNAs and 4) a small set of contributed sequences. Probe annotation was updated with OligoRAP. For the EADGENE and SABRE workshop data was provided from an experiment where the ARK-Genomics Chicken 20K array was used to determine gene expression levels in chickens 8 and 24 hours after infection with several parasites [12]. From this experiment a subset of 791 highly differentially expressed probes based on the MM8-MM24 contrast was selected for in depth analysis.

References

1. Kent WJ: **BLAT--the BLAST-like alignment tool.** *Genome Res* 2002, **12**:656-664.
2. Altschul SF, Gish W, Miller W, Myers EW, Lipman DJ: **Basic local alignment search tool.** *J Mol Biol* 1990, **215**:403-410.
3. Altschul SF, Madden TL, Schaffer AA, Zhang J, Zhang Z, Miller W, Lipman DJ: **Gapped BLAST and PSI-BLAST: a new generation of protein database search programs.** *Nucleic Acids Res* 1997, **25**:3389-3402.
4. Wheeler DL, Barrett T, Benson DA, Bryant SH, Canese K, Chetvernin V, Church DM, Dicuccio M, Edgar R, Federhen S, Feolo M, Geer LY, Helmberg W, Kapustin Y, Khovayko O, Landsman D, Lipman DJ, Madden TL, Maglott DR, Miller V, Ostell J, Pruitt KD, Schuler GD, Shumway M, Sequeira E, Sherry ST, Sirotkin K, Souvorov A, Starchenko G, Tatusov RL, Tatusova TA, Wagner L, Yaschenko E: **Database resources of the National Center for Biotechnology Information.** *Nucleic Acids Res* 2008, **36**:D13-21.
5. Flicek P, Aken BL, Beal K, Ballester B, Caccamo M, Chen Y, Clarke L, Coates G, Cunningham F, Cutts T, Down T, Dyer SC, Eyre T, Fitzgerald S, Fernandez-Banet J, Graf S, Haider S, Hammond M, Holland R, Howe KL, Howe K, Johnson N, Jenkinson A, Kahari A, Keefe D, Kokocinski F, Kulesha E, Lawson D, Longden I, Megy K, Meidl P, Overduin B, Parker A, Pritchard B, Prlic A, Rice S, Rios D, Schuster M, Sealy I, Slater G, Smedley D, Spudich G, Trevanion S, Vilella AJ, Vogel J, White S, Wood M, Birney E, Cox T, Curwen V, Durbin R, Fernandez-Suarez XM, Herrero J, Hubbard TJ, Kasprzyk A, Proctor G, Smith J, Ureta-Vidal A, Searle S: **Ensembl 2008.** *Nucleic Acids Res* 2008, **36**:D707-14.
6. Stajich JE, Block D, Boulez K, Brenner SE, Chervitz SA, Dagdigian C, Fuellen G, Gilbert JG, Korf I, Lapp H, Lehvaslaiho H, Matsalla C, Mungall CJ, Osborne BI, Pocock MR, Schattner P, Senger M, Stein LD, Stupka E, Wilkinson MD, Birney E: **The Bioperl toolkit: Perl modules for the life sciences.** *Genome Res* 2002, **12**:1611-1618.
7. Ashburner M, Ball CA, Blake JA, Botstein D, Butler H, Cherry JM, Davis AP, Dolinski K, Dwight SS, Eppig JT, Harris MA, Hill DP, Issel-Tarver L, Kasarskis A, Lewis S, Matese JC, Richardson JE, Ringwald M, Rubin GM, Sherlock G: **Gene ontology: tool for the unification of biology. The Gene Ontology Consortium.** *Nat Genet* 2000, **25**:25-29.
8. He Z, Wu L, Li X, Fields MW, Zhou J: **Empirical establishment of oligonucleotide probe design criteria.** *Appl Environ Microbiol* 2005, **71**:3753-3760.
9. **Grid Engine (GE)** [http://gridengine.sunsource.net/]
10. **Saxon-B: the Open Source SAXON XSLT and XQuery Processor.** [http://saxon.sourceforge.net/]
11. **ARK-Genomics Chicken 20K Oligo Array** [http://www.arkgenomics.org/microarrays/]
12. Hedegaard J, Bicciato S, Bonnet A, Boo MR, Buitenhuis B, Collado-Romero M, Conley LN, Cristobal MS, Ferrari F, Groenen MAM, Hornshøj H, Hulsegge I, Jiang L, Jiménez CA, Jiménez-

Marín Á, Kommadath A, Lagarrigue S, Leunissen JAM, Liaubet L, Neerincx PBT, Nie H, Pavón JJG, Prickett D, Rebel JMJ, Robert-Granié C, Skarman A, Smits MA, Sørensen P, Tosser-Klopp G, Poel Jvd, Watson M: **Methods for interpreting lists of affected genes obtained in a DNA microarray experiment.** *BMC Proceedings* 2009, **3**(Suppl 4):S5
