# Supplementary figures and images for: OligoRAP – an Oligo Re-Annotation Pipeline to improve annotation and estimate target specificity
Source: BMC Proc. 2009 Jul 16;3(Suppl 4):S4. doi: 10.1186/1753-6561-3-S4-S4 (PMC2712747; doi:10.1186/1753-6561-3-S4-S4)

# OligoRAP • Pipeline Overview

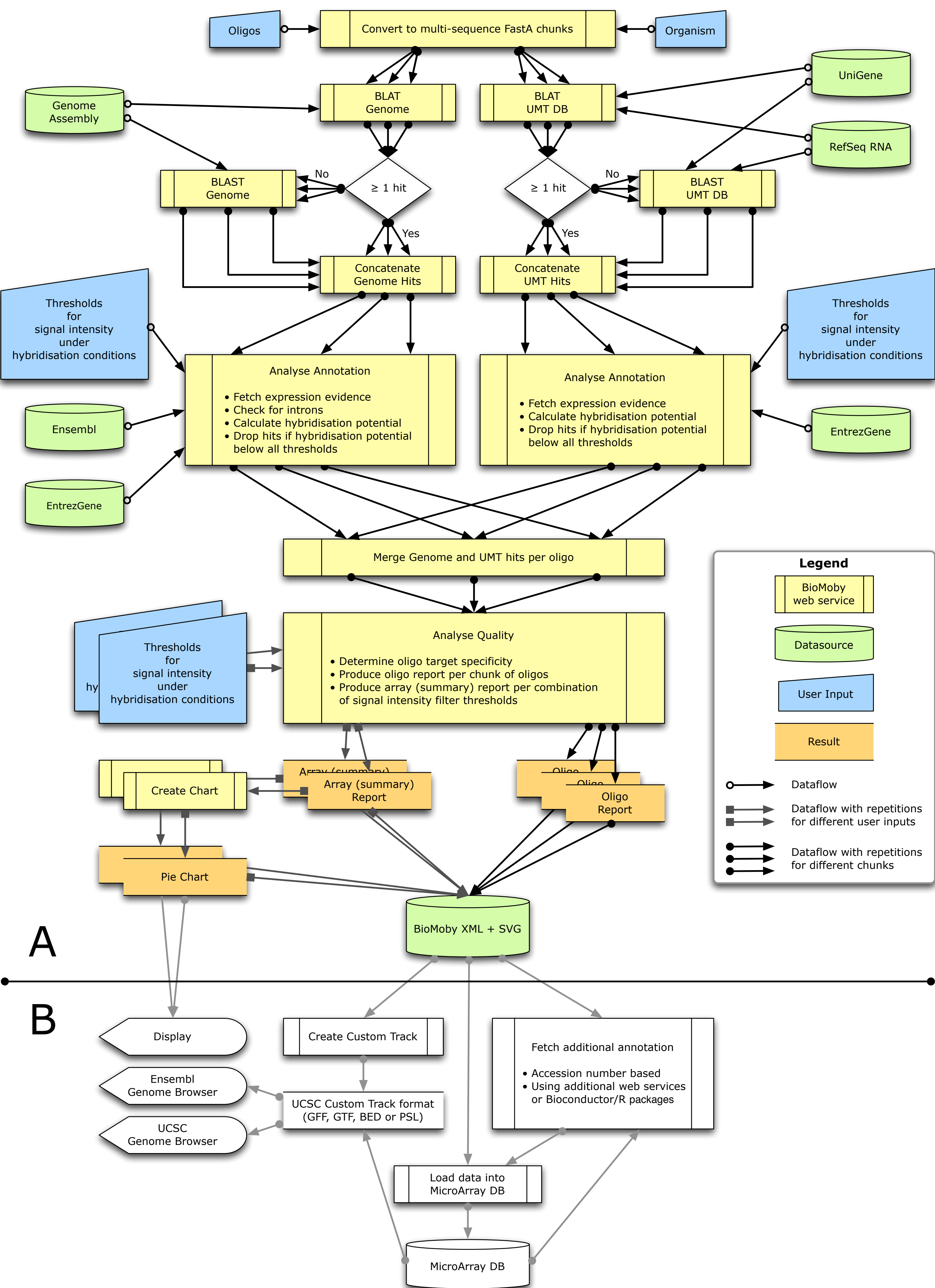

Supplement: Additional file 2 — Detailed flowchart. Figure in PDF format. (A) OligoRAP components. User inputs are in blue, databases in green and results in orange. Yellow blocks represent a single synchronous web service or a set of two asynchronous services for a specific task (one service for job submission and one for requesting a job's status). Some steps are executed multiple times. BLAT, BLAST, Concatenate, Analyse Annotation and Merge Hits and Analyse Quality are executed multiple times for multiple chunks as indicated by sets of three connecting lines starting with a filled circle (●). The Create Chart step is executed multiple times for different inputs (not chunks) as indicated by a set of two connecting lines starting with a filled square (■). (B) Some examples of how OligoRAP can be extended or linked to downstream analyses tools. [file 1753-6561-3-S4-S4-S2.pdf]

## Oligo Target Specificity Classes (TSCs)

### Target Specificity Classes

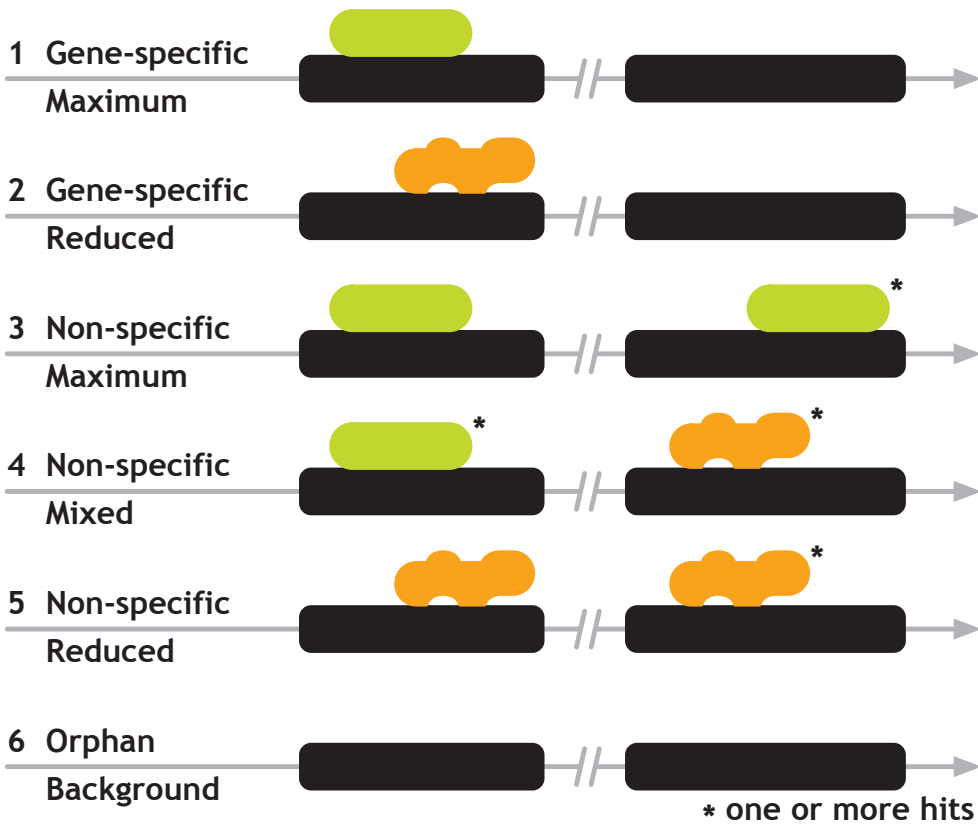

### Legend

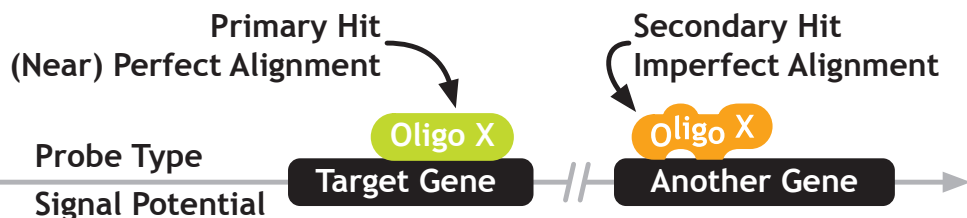

Supplement: Additional file 4 — Overview of OligoRAP's six target specificity classes, which are defined by the amount of primary and secondary hits. Figure in PDF format showing how target specificity classes are defined based on hits. Primary hits (green) represent (near) perfect alignments of oligos with their targets. Secondary hits (orange) are defined as worse than primary hits, but still capable of generating signal above background. Classes are named after the combination of probe type (gene-specific, non-specific or orphan) and estimated potential signal (maximum, reduced, mixed or background). [file 1753-6561-3-S4-S4-S4.pdf]
