# Supplementary material for: OligoRAP – an Oligo Re-Annotation Pipeline to improve annotation and estimate target specificity
Source: BMC Proc. 2009 Jul 16;3(Suppl 4):S4. doi: 10.1186/1753-6561-3-S4-S4 (PMC2712747; doi:10.1186/1753-6561-3-S4-S4)

Filter Thresholds versus Estimated Signal

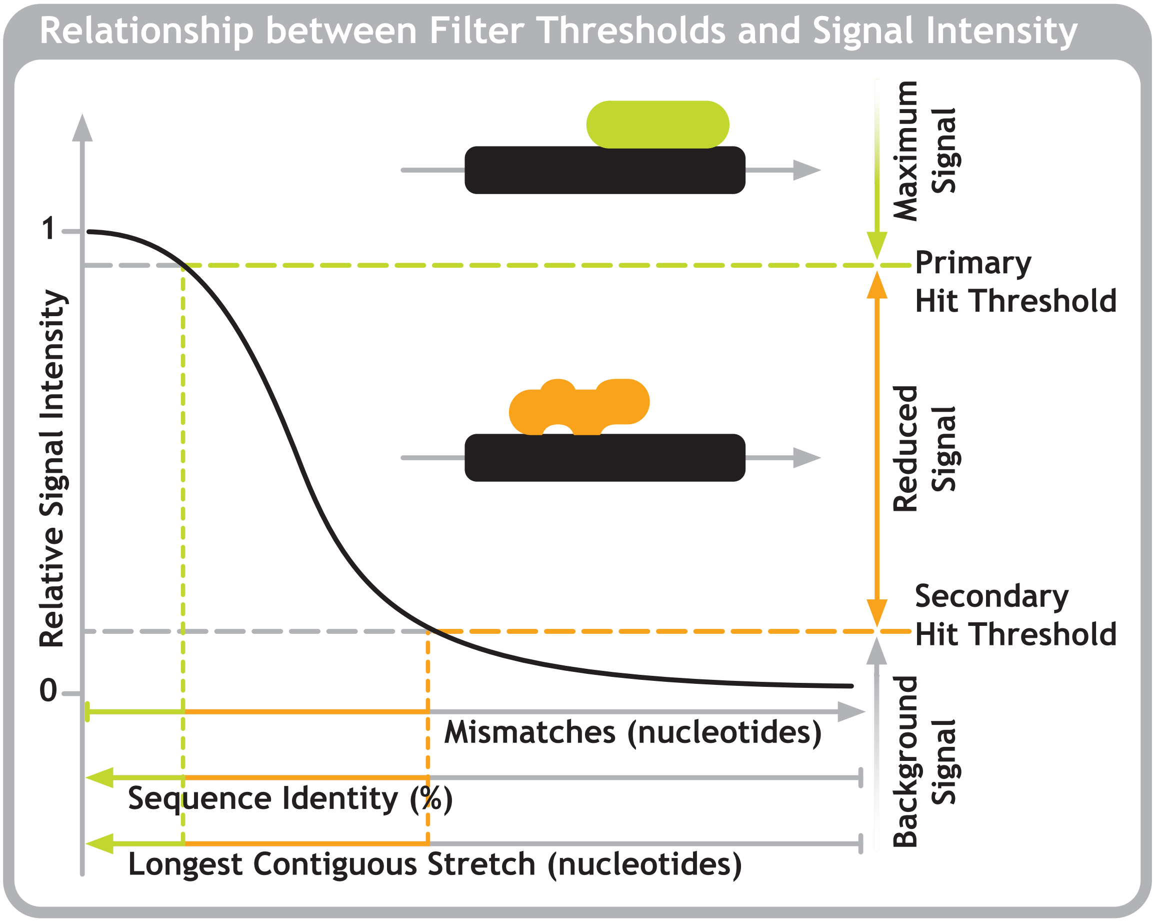

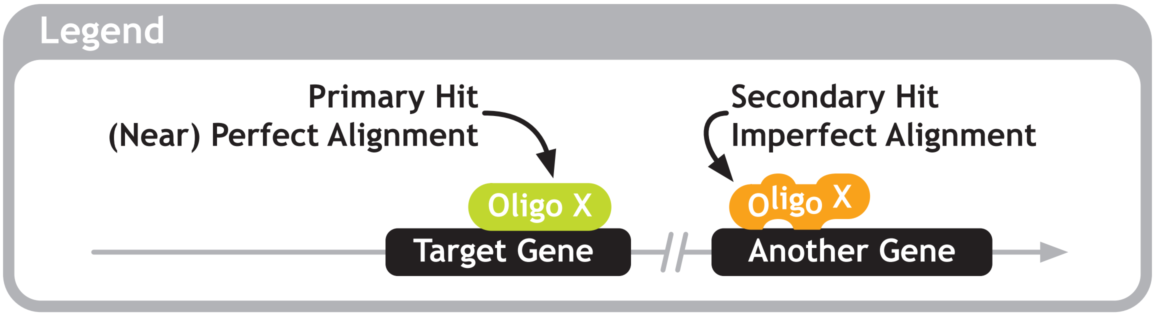

Supplement: Additional file 3 — Relationships between filter thresholds, primary & secondary hits and estimated signal intensity. Figure in PDF format. Primary hits (green) represent (near) perfect alignments of oligos with their targets. Secondary hits (orange) are defined as worse than primary hits, but still capable of generating signal above background. Relative signal intensity is shown on the vertical axis and the 3 filters – mismatches, sequence identity and longest contiguous stretch – on 3 horizontal axes. Signal intensity drops as the amount of mismatches increases and as the percentage sequence identity or the length of the longest contiguous stretch decreases. Estimated signal intensity above the primary hit threshold (green) is defined as "maximum signal". Estimated signal below the primary and above the secondary hit threshold (orange) is defined as "reduced". Finally estimated signal below the secondary hit threshold is defined as "background signal". [file 1753-6561-3-S4-S4-S3.pdf]
