# Supplementary material for: OligoRAP – an Oligo Re-Annotation Pipeline to improve annotation and estimate target specificity
Source: BMC Proc. 2009 Jul 16;3(Suppl 4):S4. doi: 10.1186/1753-6561-3-S4-S4 (PMC2712747; doi:10.1186/1753-6561-3-S4-S4)
Supplement: Additional file 5 — OligoQualityAnalyser example output for Compugen oligo CGEN-MOUSE_30000003_1. Figure in PDF format showing an example of OligoQualityAnalyser output with an OligoQualityRecord (blue) containing 2 hits/alignments (green) and 2 target specificity assignments (orange). The first hit overlaps with Ensembl features resulting in annotation in the form of cross-references (purple), while the second hit targets 'intergenic' space resulting in a lack of cross-references. Each target specificity block contains the oligo's specificity for two contexts – genome and transcriptome – and refers by id attribute to the filter settings (thresholds) described elsewhere in the XML (not shown). The Cigar Like Line (CLL) is derived from the Ensembl Cigar line [21] and is used to store alignment details (matches, mismatches, insertions, deletions & intron gaps) in a compact string. See figure additional file 6 for detailed CLL examples. Together with subject sequence accession/ID, start, and stop, the CLL provides all information necessary to reconstruct the alignments. It can be used for example to create UCSC "custom tracks" [22,23] for visualization in the UCSC or Ensembl genome browsers. [file 1753-6561-3-S4-S4-S5.pdf]

OligoQualityAnalyser example output for an oligo

BioMoby XML


```xml
<?xml version='1.0' encoding='UTF-8'?>
<moby:MOBY xmlns:moby='http://www.biomoby.org/moby' xmlns='http://www.biomoby.org/moby'>
  <moby:mobyContent moby:authority='www.bioinformatics.nl/phenolink/'>
    <moby:mobyData moby:queryID='1146'>
      <moby:Collection moby:id='CGEN_MOULIB384' moby:namespace='' moby:articleName='oligo_annotation'>
        <moby:Simple moby:articleName='oligo' moby:id='1'>
          <moby:OligoQualityRecord moby:id='CGEN_MOUSE_3000003_1|NM_008954' moby:namespace=''
              moby:articleName='oligo_record'>
            <moby:Integer moby:id='' moby:namespace='' moby:articleName='length'>65</moby:Integer>
            <moby:OligoHit moby:id='hit1 hsp1' moby:namespace='Mus_musculus_Genome' moby:articleName='oligo_hit'>
              <moby:HitPosition moby:id='' moby:namespace='transcriptome' moby:articleName='hit_position'>
                <moby:String moby:id='' moby:namespace='' moby:articleName='seq_id'>17</moby:String>
                <moby:Integer moby:id='' moby:namespace='' moby:articleName='start'>57139132</moby:Integer>
                <moby:Integer moby:id='' moby:namespace='' moby:articleName='stop'>57139196</moby:Integer>
                <moby:Object moby:id='-' moby:namespace='' moby:articleName='strand'/>
                <moby:String moby:id='' moby:namespace='' moby:articleName='cll'>65M</moby:String>
              </moby:HitPosition>
              <moby:CrossReference>
                <moby:Object moby:id='0001658' moby:namespace='GO' moby:articleName='annotation_xref'/>
                <moby:Object moby:id='0005102' moby:namespace='GO' moby:articleName='annotation_xref'/>
                <moby:Object moby:id='0005576' moby:namespace='GO' moby:articleName='annotation_xref'/>
                <moby:Object moby:id='0005615' moby:namespace='GO' moby:articleName='annotation_xref'/>
                <moby:Object moby:id='0008083' moby:namespace='GO' moby:articleName='annotation_xref'/>
                <moby:Object moby:id='19197' moby:namespace='EntrezGene' moby:articleName='expression_xref'/>
                <moby:Object moby:id='A1L3Q1' moby:namespace='UniProt' moby:articleName='expression_xref'/>
                <moby:Object moby:id='AF040960' moby:namespace='INSDC' moby:articleName='expression_xref'/>
                <moby:Object moby:id='BC130229' moby:namespace='INSDC' moby:articleName='expression_xref'/>
                <moby:Object moby:id='ENSBTAG00000031794' moby:namespace='Ensembl'
                  moby:articleName='Bos_taurus_homology_xref'/>
                <moby:Object moby:id='ENSG00000125650' moby:namespace='Ensembl'
                  moby:articleName='Homo_sapiens_homology_xref'/>
                <moby:Object moby:id='ENSMUSG00000002664' moby:namespace='Ensembl'
                  moby:articleName='expression_xref'/>
                <moby:Object moby:id='ENSMUST00000002740' moby:namespace='Ensembl'
                  moby:articleName='expression_xref'/>
                <moby:Object moby:id='GENSCAN00000045225'
                  moby:namespace='in silico' moby:articleName='expression_xref'/>
                <moby:Object moby:id='Mm.86487' moby:namespace='UniGene.cluster'
                  moby:articleName='expression_xref'/>
                <moby:Object moby:id='NM_008954' moby:namespace='RefSeq' moby:articleName='expression_xref'/>
                <moby:Object moby:id='O70300' moby:namespace='UniProt' moby:articleName='expression_xref'/>
              </moby:CrossReference>
            </moby:OligoHit>
            <moby:OligoHit moby:id='hit2 hsp1' moby:namespace='Mus_musculus_Genome' moby:articleName='oligo_hit'>
              <moby:HitPosition moby:id='' moby:namespace='genome' moby:articleName='hit_position'>
                <moby:String moby:id='' moby:namespace='' moby:articleName='seq_id'>11</moby:String>
                <moby:Integer moby:id='' moby:namespace='' moby:articleName='start'>3259678</moby:Integer>
                <moby:Integer moby:id='' moby:namespace='' moby:articleName='stop'>3259742</moby:Integer>
                <moby:Object moby:id='+' moby:namespace='' moby:articleName='strand'/>
                <moby:String moby:id='' moby:namespace='' moby:articleName='cll'>15S16M34S</moby:String>
              </moby:HitPosition>
            </moby:OligoHit>
            <OligoTargetSpecificity moby:namespace='' moby:id='fs1' moby:articleName='oligo_target_specificity'>
              <Object moby:namespace='' moby:id='1p:0s' moby:articleName='transcriptome'/>
              <Object moby:namespace='' moby:id='1p:0s' moby:articleName='genome'/>
            </OligoTargetSpecificity>
            <OligoTargetSpecificity moby:namespace='' moby:id='fs2' moby:articleName='oligo_target_specificity'>
              <Object moby:namespace='' moby:id='1p:0s' moby:articleName='transcriptome'/>
              <Object moby:namespace='' moby:id='1+p:1+s' moby:articleName='genome'/>
            </OligoTargetSpecificity>
          </moby:OligoQualityRecord>
        </moby:Simple>
      </moby:Collection>
    </moby:mobyData>
  </moby:mobyContent>
</moby:MOBY>
```


Legend

BioMoby header/footer | One oligo | Two hits | Two TSC assignments | Annotation/Xrefs
