# Supplementary material for: OligoRAP – an Oligo Re-Annotation Pipeline to improve annotation and estimate target specificity
Source: BMC Proc. 2009 Jul 16;3(Suppl 4):S4. doi: 10.1186/1753-6561-3-S4-S4 (PMC2712747; doi:10.1186/1753-6561-3-S4-S4)
Supplement: Additional file 6 — Detailed CLL examples. Figure in PDF format showing five example alignments with their accompanying cigar like lines (CLLs). A CLL is a compact way to represent the alignment of a first sequence (query) with a second one (subject/DB). In these examples the single stranded oligo is the query and the double stranded DNA the subject. Example with matches and substitutions of an oligo hit on the forward DNA strand (1). Similar example of an oligo hit on the reverse DNA strand (2). Note that the CLL describes the alignment from the perspective of the oligo in terms of insertions and deletions, but is always read from left to right with the forward strand of the subject written from 5' on the left to 3' on the right side. Hence in the case of example 2 the CLL corresponds to 3' on the left to 5' on the right for the oligo sequence. Examples of insertions & deletions (3) and of an intron gap (4). In this context introns are special cases of deletions and usually the result of merging multiple smaller hits into one larger alignment. Example of mixed case nucleotides (5): a number followed by 2 or more characters (m/s/i/d/n) indicates this amount of nucleotides can be a mix of the corresponding classes. In this case there are 25 ns nucleotides, which corresponds to a mix of substitutions with an intron gap. Due to the substitutions it's not possible to determine exactly where the intron gap starts and ends in the oligo sequence. Hence alignments corresponding to 7m5s20n9m, 7m3s20n2s9 m (shown) and 7m20n5s9 m can al be written as 7m25ns9m. [file 1753-6561-3-S4-S4-S6.pdf]

Detailed cigar-like line (CLL) examples

Alignments with their corresponding CLLs

```
1. Oligo + 5' gtaaggcacagaaggtggcagcaagtctgtcggtgatca 3'
                     ||||||||||||||||||    ||||||||||     CLL 7s18m4s10m
   DNA   + 5' tggctttacagaaggtggcagcaagaacatcggtgatca 3'
   DNA   - 3' accgaaatgtcttccaccgtcgttcttgtagccactagt 5'


2. DNA   + 5' tggctttacagaaggtggcagcaagaacatcggtgatca 3'
   DNA   - 3' accgaaatgtcttccaccgtcgttcttgtagccactagt 5'
                     ||||||||||||||||||    ||||||||||     CLL 7s18m4s10m
   Oligo + 3' gtgaggttgtcttccaccgtcgttcagacagccactagt 5'


3. Oligo + 5' gtaaggcacagaaggtggcagcaag-----cggtgatca 3'
                     |||||||  |||||||||     |||||||||     CLL 7s7m2i9m5d9m
   DNA   + 5' tggctttacagaag--ggcagcaagaacatcggtgatca 3'
   DNA   - 3' accgaaatgtcttc--ccgtcgttcttgtagccactagt 5'


4. Oligo + 5' tggcttt-----------------------cggtgatca 3'
              |||||||                       |||||||||     CLL 7m23n9m
   DNA   + 5' tggctttacagaaggtggcagcaagaacatcggtgatca 3'
   DNA   - 3' accgaaatgtcttccaccgtcgttcttgtagccactagt 5'


5. Oligo + 5' tggctttgat-------------------cgcggtgatca 3'
              |||||||                       |||||||||    CLL 7m23ns9m
   DNA   + 5' tggctttacagaaggtggcagcaagaacatcggtgatca 3'
   DNA   - 3' accgaaatgtcttccaccgtcgttcttgtagccactagt 5'
```

Legend

M = Matches **S = Substitutions** **I = Insertions** **D = Deletions** **N = iNtrons (Special case of D)**
